# Genetics of Sub-Saharan African Human Population: Implications for HIV/AIDS, Tuberculosis, and Malaria

**DOI:** 10.1155/2014/108291

**Published:** 2014-08-18

**Authors:** Gerald Mboowa

**Affiliations:** ^1^Department of Medical Microbiology, College of Health Sciences, Makerere University, P.O. Box 7072, Kampala, Uganda; ^2^School of Allied Health Sciences, International Health Sciences University, P.O. Box 7782, Kampala, Uganda

## Abstract

Sub-Saharan Africa has continued leading in prevalence and incidence of major infectious disease killers such as HIV/AIDS, tuberculosis, and malaria. Epidemiological triad of infectious diseases includes susceptible host, pathogen, and environment. It is imperative that all aspects of vertices of the infectious disease triad are analysed to better understand why this is so. Studies done to address this intriguing reality though have mainly addressed pathogen and environmental components of the triad. Africa is the most genetically diverse region of the world as well as being the origin of modern humans. Malaria is relatively an ancient infection in this region as compared to TB and HIV/AIDS; from the evolutionary perspective, we would draw lessons that this ancestrally unique population now under three important infectious diseases both ancient and exotic will be skewed into increased genetic diversity; moreover, other evolutionary forces are also still at play. Host genetic diversity resulting from many years of malaria infection has been well documented in this population; we are yet to account for genetic diversity from the trio of these infections. Effect of host genetics on treatment outcome has been documented. Host genetics of sub-Saharan African population and its implication to infectious diseases are an important aspect that this review seeks to address.

## 1. Introduction

HIV/AIDS, TB, and malaria are three important killer diseases globally; however, these continue to devastate more of sub-Saharan African populations, with a total of 27 countries. Sub-Saharan Africa alone accounted for an estimated 70 percent of all people living with HIV [1] and 70 percent of all AIDS deaths in 2011 [2]. Data published by the World Health Organization (WHO 2014, http://www.who.int/mediacentre/factsheets/fs104/en/) indicate that sub-Saharan Africa carried the greatest proportion of new tuberculosis cases per population with over 255 cases per 100 000 population in 2012. (WHO 2013 http://www.who.int/mediacentre/factsheets/fs094/en/) indicate most malaria cases and deaths occur in sub-Saharan Africa.

Sub-Saharan Africa is important regarding origin of human species and the way in which genetic variation affects human phenotypes. Africa is thought to be the ancestral homeland of all modern humans and is the most recent homeland of millions of individuals whose ancestors were brought to Europe and to the Americas as slaves [3]. There is much to be learned from the genetics of sub-Saharan African populations about human origins and evolution and about the origin and nature of human complex disease. At present, we have little understanding of the genetic structure of sub-Saharan populations and the genetic basis of complex disease in African populations because very few genetic studies have been conducted in African ethnic groups. Research activity has traditionally been biased towards the study of non-African populations, and our knowledge of even the most fundamental information about the genetic basis of disease in Africa is quite limited [3] yet these three infectious diseases account for the highest morbidity and mortality. Increased funding and resources for studying genetic diversity in Africa are needed to reconstruct human evolutionary history, to dissect the genetic basis of resistance and susceptibility to disease, and to design better drugs for all people [3]. I suggest that this will require collaborative efforts with African institutions and ensure that the capacity is sustained. A shared set of DNA resources and the establishment of an African genetic database would help to provide researchers with common information and would facilitate studies of several loci in the same set of African populations. Studies also need to meet strict ethical standards and to involve both local researchers and study participants [3]. I am compelled to propose that modern humans who migrated away from sub-Saharan Africa encountered new environment and exotic pathogens in their new geographical niches. Those exotic pathogens and other evolutionary forces, which they met in the different areas where they settled, would account for unique genetics. Infectious diseases have continued and will continue shaping the course of human evolution.

The hypothesis whereby infectious diseases have been acting as a powerful selective pressure was formulated long ago, but it was not until the availability of large-scale genetic data and the development of novel methods to study molecular evolution that we could assess how pervasively infectious agents have shaped human genetic diversity [4]. Disease outcome is multifactorial process, requiring interplay of host-environment-microbial factors which ultimately determine disease resistance and progression. Genetic structures of the exposed human populations will determine the susceptibility patterns that are always observed in the host. Recent genome-wide analysis indicates that among the diverse environmental factors that most likely acted as selective pressures during the evolution of human species (climate, diet regimes, and infections) pathogen load had the strongest influence on the shaping of human genetic variability [5]. Possibly the indigenous pathogens in sub-Saharan Africa coevolved with their hosts creating unique genetic profiles in these human populations. I propose that a form of Newton's third law of motion happens during an interaction between host and pathogen; action and reaction are equal and opposite. This implies that the selective pressure exerted by these pathogens onto selected host genes in response to specific pathogen genes received similar pressure from the host driving host-pathogen diversity observed as unique genetic profiles in both host and pathogen accounting for coevolution. Studies show certain host-pathogens specificities, probably due to coevolution. I further propose that these unique genetic profiles created over time affect vaccine efficacy and of late we know that treatment outcome is also affected by the host genetic structures. The unique genetic profiles created in these human populations can act as risk genetic factors for emerging pathogens. Penicillin was a wonder drug at the birth of the antibiotic era but after half a century of its clinical use, we observe emergence of penicillin resistant bacteria strains; these have acquired genetic variations that their ancestors never had. In the antibiotic/antiviral era, drugs are also likely to act as selective pressure creating genetic diversity in pathogen genomes as well as hosts'.

## 2. Human Host Genetic Diversity and Infectious Diseases

The high levels of genetic diversity in African populations and their demographic history make these populations particularly informative for the fine mapping of complex genetic diseases [6] as well as infectious diseases. Studies using mitochondrial (mt) DNA and nuclear DNA markers consistently indicate that Africa is the most genetically diverse region of the world [7]. Historically, human population genetic studies have relied on one or two African populations as being representative of African diversity, but recent studies show extensive genetic variation among even geographically close African populations, which indicates that there is not a single “representative” African population [3].

The immunological responses to MTB are due to the interaction between the immune system, human host, and bacterial and environmental factors [8]. Genetic as well as acquired defects in host immune response pathways greatly increase the risk of progressive disease [9]. Results from genome-wide linkage studies suggest that TB disease susceptibility is highly likely to be polygenic, with contributions from many minor loci [10], and a large number of TB susceptibility markers have been identified from candidate gene studies as “disease-causing” genes which include TIRAP, HLA DQB1, VDR, IL-12*β*, IL12R*β*1, IFN-*γ*, SLC11A1, and MCP-1. However, to date the greatest evidence to support an underlying genetic basis for TB has come from the discovery of single gene defects predisposing to disseminated and often lethal mycobacterial disease [11].

To better understand why HIV/AIDS, tuberculosis, and malaria are more common infectious diseases in sub-Saharan Africa, we must appreciate the region's human genetic diversity and this will provide data that may help understand why there is a mixed population of both resistant and susceptible populations coexisting. We can assert that the indigenous infections like malaria and tuberculosis have created unique genetic structures in these mixed ethnic populations which can be risk factors for exotic infectious diseases like HIV/AIDS and vice versa. A notion that exposure to indigenous pathogens/parasites in these areas shaped the genetic structures of these native human populations resulting in interethnic variation in susceptibility to modern infectious agents is undisputable. The great human genetic diversity and the continued presence of infectious agents in sub-Saharan Africa will continue contributing to the observed susceptibilities to infectious diseases.

## 3. HIV/AIDS

Host genetic polymorphisms contribute to interindividual variation in susceptibility to acquiring HIV-1 infection, the degree of infectiousness to others (as reflected by the viral load), and rates of disease progression [12]. Such polymorphisms might also contribute to significant interpopulation differences in HIV-1 prevalence [12–16]. Duffy antigen receptor for chemokines (DARC) expressed on red blood cells (RBCs) influences plasma levels of HIV-1-suppressive and proinflammatory chemokines such as CCL5/RANTES [17]. DARC is also the RBC receptor for* Plasmodium vivax*. Africans with* DARC*-*46C/C* genotype, which confers a DARC- negative phenotype, are resistant to* vivax malaria*. HIV-1 attaches to RBCs via DARC, effecting transinfection of target cells [17]. In African Americans,* DARC-46C/C* is associated with 40% increase in the odds of acquiring HIV-1 [17]. If extrapolated to Africans, about 11% of the HIV-1 burden in Africa may be linked to this genotype. After infection occurs, however, DARC-negative RBC status is associated with slower disease progression [17].

Host genetic influence on HIV/AIDS susceptibility by a polymorphism that is differentially distributed between populations is best illustrated by the effects of the European-specific 32 bp deletion (D32) in the gene for CC chemokine receptor 5 (CCR5), which is the major coreceptor for R5 strains of HIV-1 that represent the majority of transmissible viruses [18]. CCR5-Δ32 exhibit variable frequencies in distinct populations [19–22] and possibly their phenotypes depend on the host ethnicity analyzed [12, 20–24]. Distribution of the protective Δ32 allele is however restricted to Northern Europe, where it occurs at a frequency of (10–16)% and its frequency decreases in a Southeast cline towards the Mediterranean and gradually disappears in the African and Asian populations [25]. The Δ32 mutation is most common in individuals of European descent but is absent or rare in the black African population [25]. A recent study in South Africa detected Δ32 mutation only in the Caucasian group and it also reported greater genetic diversity in CCR5 gene in the black South African population showing 39 mutations being exclusive [26]. This extensive variation in CCR5 gene in a small studied African population is in agreement with the concept that Africans have the greatest ethnic genetic diversity in the world.

The genes CCL3L1/CCL4L1 encode the chemokines MIP-1*α* and MIP-1*β* which are both ligands for the chemokine receptor CCR5 used as a coreceptor by R5 strains of HIV [27].* CCL3L1* gene dose has been associated with intersubject differences in susceptibility to HIV acquisition in European, African, and Hispanic-American adults, intravenous drug users from Estonia, and hemophiliacs from Japan [13, 28, 29]. The average copy number of* CCL3L1 *varies among populations [13]. A study in Central African Pygmies indicated that there might be a CCL3L1-CCR5-dependent biological basis for interpopulation differences in HIV prevalence and concluded that the copy number of* CCL3L1 *genes is determinant of HIV-AIDS susceptibility [30].

The CCR5-Δ32 mutation along with single nucleotide polymorphisms (SNPs) in the CCR5 promoter and the CCR2-V64I mutation have been included in seven human haplogroups (HH) previously associated with resistance/susceptibility to HIV-1 infection and different rates of AIDS progression [31].* In vitro *studies have demonstrated that HIV-1 can use alternative coreceptors, such as CCR2 [32]. A genetic variant of CCR2 (CCR2-V64I) leads to variations in the CCR2 transmembrane region and has been associated with slow progression to AIDS; however, its effect on susceptibility to acquire HIV-1 infection has not been defined to date [33]. This would be a study worth carrying out in sub-Saharan African population. Recently, the immunoregulatory cytokine IL-10 was identified as playing a key role in suppressing antiviral immune responses, leading to viral persistence [34, 35]. A study in South Africa populations indicated that individuals carrying the IL-10-592AA genotype were more likely to become HIV-1 infected further; these results generally suggested that IL-10 promoter polymorphisms were linked to low IL-10 production and associated with increased HIV-1 susceptibility [36].

A study in Kenya revealed pathological interactions between HIV and malaria in dually infected patients, but the public health implications of the interplay have remained unclear [37]. A transient almost one-log elevation in HIV viral load occurred during febrile malaria episodes; in addition, susceptibility to malaria was enhanced in HIV-infected patients [37]. Coinfection might also have facilitated the geographic expansion of malaria in areas where HIV prevalence is high; therefore, transient and repeated increases in HIV viral load resulting from recurrent coinfection with malaria may be an important factor in promoting the spread of HIV in sub-Saharan Africa [37].

## 4. Tuberculosis

A lot of attention has been given to study the importance of the* Mycobacterium tuberculosis *pathogen and the genetic constitution of the host largely ignored especially in the most affected regions like sub-Saharan Africa. It is estimated that only 10% of those who become infected with* Mycobacterium tuberculosis *will ever develop clinical disease [38]. A growing body of evidence suggests that host genetics play a role in the predisposition to tuberculosis (TB) disease, in addition to pathogen, environmental, and socioeconomic factors [39, 40]. Genetic factors contributing to TB susceptibility include variants of the human leukocyte antigen (HLA) class II complex [41–44] and the vitamin D receptor gene (*VDR*) [45–48]. HLA alleles are found to be associated with susceptibility and resistance to infectious diseases including HIV/AIDS, tuberculosis, and malaria that impose huge public health burdens in sub-Saharan Africa [49, 50]. HLA studies have also yielded important insights into the role of pathogens in driving HLA polymorphism. For example, a study that analyzed 61 human populations across the world showed that populations that have a greater burden of pathogens show higher HLA diversity and those populations farther from Africa (geographic distance measured through landmasses from Ethiopia) are characterized by lower HLA diversity [51].

Tuberculosis was a major selective force in the evolution of Western European populations, whereas malaria served a similar role in Africa [52, 53]. Genes involved in protective immunity are under greater selective pressure, showing greater variability than other genes [52, 53]. For a disease to be a selective pressure in the evolution of a human population, the gene would have to have a significant impact for long periods of time, influencing morbidity and mortality before reproductive age [52, 53]. TB is currently a world-wide pathogen, and archeologic evidence indicates a great prehistoric prevalence for the disease in crowded cities of Europe and North Africa [54, 55]. It appears, however, that this organism was once completely absent from several isolated areas [56, 57], the largest of which was Africa [58]. Recent observations strongly suggest a significant role for genetic factors in innate resistance to infection by* Mycobacterium tuberculosis *[58]. This relation was discovered in a study of tuberculosis in Arkansas nursing homes and was supported by data from three outbreaks of tuberculosis in two prisons [58]. A person's resistance level was found to correlate with the region of his or her ancestry [58]. Ancestors of persons in the more resistant group tended to derive from densely populated areas and cities rife with tuberculosis, whereas the ancestors of persons in the more susceptible group tended to derive from areas once free of tuberculosis [58].

With the completion of Human Genome Project and advances in genotyping technology, Genome-Wide Association (GWA) Study has been one powerful tool for the study of genetic susceptibility in human complex diseases [59]. Recently a study conducted in Ghana and The Gambia using Genome-Wide Association Study (GWAS) identified a susceptibility locus of rs4331426 on chromosome 18q11.2 for tuberculosis in the African population [60]. In a case-control study in The Gambia, two candidate genes, natural resistance associated macrophage protein (*NRAMP1)* and* VDR*, were found to be associated with tuberculosis [46, 61]. Another study in The Gambia and South Africa reported using a genome-wide search on a large number of families with an infectious disease results from linkage analysis alone provided suggestive evidence for tuberculosis-susceptibility genes on chromosomes 15q and Xq, and these results were supported by the results of common ancestry using microsatellite (CAM) mapping [62].

Macrophage migration inhibitory factor (MIF) is an innate cytokine encoded in a functionally polymorphic genetic locus [63]. The number of CATT tetranucleotide repeats at −794 is associated with differential MIF expression: the −794 CATT5 variant is a low-expression allele [63]. Globally, the CATT5 allele is more frequently identified among African-Americans and Africans compared with their Caucasian American or Western European counterparts [64]. The proposed MIF-TB susceptibility allele (−794 CATT5) is found commonly in Caucasians (~45%) and is more prevalent in African-Americans and in Africans (60–80%), indicating that MIF genotype may make a clinically meaningful contribution to TB disease risk [64, 65]. The results from a recent study in Uganda found that mycobacteremic subjects were more likely to be genotypic low expressers of MIF (−794 CATT5/5, 33% versus 18%, odds ratio (OR) 2.2, *P* = 0.009). This association remained significant in multivariate analysis adjusting for age, sex, CD4 count, and use of highly active antiretroviral therapy (HAART) [63].

Studies by Khor et al., looking at variant alleles of* CISH* gene polymorphisms and susceptibility to infectious diseases found that the minor alleles of three SNPs (−292, +1320, and +3415) were associated with increased susceptibility to TB in Malawian population and further reported that TB susceptibility in the Gambia was accounted for by more than one SNP, implying a highly genetically diverse population [66].

## 5. Malaria

During the course of human evolution in regions where malaria is prevalent, naturally occurring genetic defense mechanisms have evolved for resisting infection by plasmodium [67]. Most of the human genes that are thought to provide reduced risk from malarial infection are expressed in red blood cells or play a role in the immune system and they include human leukocyte antigen (HLA), *α*- and *β*-globin, Duffy factor (*FY)*, tumor necrosis factor (*TNF)*, *α* and glucose-6-phosphate dehydrogenase (*G6PD*) [67]. Perhaps no disease in history has exerted as strong a selective pressure on the human genome as* falciparum malaria* [67]. Evidence suggests that* Plasmodium falciparum *has infected humans for at least 5000–10,000 years, and human haplotype studies have shown that alleles that may offer protection against malaria have undergone selection during that same time frame [68, 69]. The selection pressure from malaria has led to many variants of erythrocytes other than hemoglobin S (HbS) [70].

Anatomically modern humans appeared in East Africa about 200,000 years ago, spread out from sub-Saharan Africa approximately 100,000 years ago, and subsequently colonized the rest of the world in a series of migratory events [71]. This implies that ethnic populations that remained in sub-Saharan Africa have been exposed to malaria for periods long enough to shape their genetic structures by plasmodium unlike those that left this region around that time. Geographical, epidemiological, and* in vitro *evidence support the hypothesis that G6PD enzyme deficiency confers protection from disease caused by the* Plasmodium falciparum *parasite [72]. G6PD is encoded by a 16.2 kb gene found on the X chromosome and approximately 160 genetic variants causing clinical deficiency of G6PD have been characterized [73]. The geographical distribution of these deficiency alleles closely reflects human populations exposed historically to endemic malaria [74]. Relatively little attention has been given to the associations between genetic polymorphisms and uncomplicated malaria, which accounts for over 99% of malarial illnesses in countries where malaria is endemic [75]. The 202A/376G G6PD A-allele is considered to be the most common G6PD deficiency allele in sub-Saharan Africa [76], though incidence and prevalence of malaria have continued to be high in this region yet known to confer protection against malaria. We suggest that protection may be conferred by more than one gene; concept remains to be elucidated.

In western and central Africa, 95%–99% of humans have the Duffy negative phenotype, a condition that is thought to confer complete protection against the* Plasmodium vivax *during the blood stages of its life cycle [77, 78]; however,* Plasmodium falciparum *is known to be the commonest cause of malaria in Africa making this phenotype less useful in sub-Saharan African population. A study on human genetic polymorphisms and asymptomatic* Plasmodium falciparum *malaria in Gabonese school children did not find statistically significant association between mannose binding lectin (MBL), tumor necrosis factor-*α* (TNF-*α*), and nitric oxide synthase 2(NOS2) polymorphisms and asymptomatic malaria [79]. Reports associating several genetic disorders with malaria susceptibility or resistance are on the rise, and studies of heritability indicate that approximately 25% of the risk for severe malaria progression is determined through human genetic factors [80]. The genetic background of the affected individual might also influence cytokine expression and disease outcomes [81, 82]. Notably, the frequency of genetic alterations differs depending on the population origin and structure, and some mutations might differentially influence the disease outcome in different patterns [80]. However, heritability studies have suggested that although about 25% of total variation in malaria incidence and hospitalization can be accounted for by host genetic variation, sickle cell status accounts for only 2% of this variation [83]. Thus, many other genetic factors likely affect the outcome studied by Crompton et al. [83]. Moreover, specific genetic factors will likely affect different populations differently, and certain polymorphisms (e.g., absence of the Duffy antigen, which protects against* Plasmodium vivax *infection primarily in Africans, and the band 3 mutation that causes ovalocytosis, primarily in Asian populations) have strong geographic determinants [68].

## 6. Concluding Remarks

Infectious diseases continue to present a major threat for human populations and, consequently, shape genetic diversity of human populations. In the western world, application of genetic and genomic technologies to study human genetic basis of diseases has been widely performed; however, in sub-Saharan Africa, where infectious diseases remain an important component of human survival, these studies are limited in sub-Saharan Africa. A lot of research has been done to describe pathogen and environment factors associated with the high prevalence and incidence of these diseases in sub-Saharan Africa; however, less attention has been given to human host genetic factors in sub-Saharan Africa population largely due to limited expertise in Africa scholars and the technologies. This has been further complicated by the fact that more than 40% of African scientists live in Organization for Economic Cooperation and Development (OECD) countries, predominately in Europe, the United States, and Canada [83]. In some OECD countries, like the United States, sub-Saharan Africans are the most educated immigrant group [84]. Therefore, African institutions should embrace genetic and genomic studies so that they can provide meaningful data on the genetic basis of infectious diseases in sub-Saharan Africa human population. This will guide new drug and vaccine development. This will increase our understanding of the human genetic markers responsible for the observed variations in susceptibilities to infectious diseases in human populations in sub-Saharan Africa.

Genetics can affect treatment outcomes, as was demonstrated in a recent study that showed sulfadoxine-pyrimethamine treatment to be approximately half as likely to fail in children with sickle cell trait, compared with children who had normal hemoglobin [85]. This further may require that regions embrace individualized medicines, since even in the proantibiotic/proantiviral and vaccine era, no much significant success has been registered to eliminate these infectious diseases. Host genetic diversity in this region may need to be matched with customized medicines. This will need collaborations and commitment from Africans at various levels. We are optimistic that genetic and genomic studies on large sub-Saharan African population will provide a great deal of insight into why the three important global infectious diseases are more prevalent in this region irrespective of available interventions and further guide new treatment interventions and vaccine development.
